# Stability of Polymeric Membranes to UV Exposure before and after Coating with TiO_2_ Nanoparticles

**DOI:** 10.3390/polym14010124

**Published:** 2021-12-30

**Authors:** Geórgia Labuto, Sandra Sanches, João G. Crespo, Vanessa J. Pereira, Rosa M. Huertas

**Affiliations:** 1 Department of Chemistry, Universidade Federal de São Paulo, Diadema 09913-030, Brazil; 2 Laboratory of Integrated Sciences—LabInSciences, Universidade Federal de São Paulo, Diadema 09913-030, Brazil; 3 iBET, Instituto de Biologia Experimental e Tecnológica, Apartado 12, 2781-901 Oeiras, Portugal; sandramsanches@gmail.com (S.S.); vanessap@ibet.pt (V.J.P.); rosa.huertas@ibet.pt (R.M.H.); 4 LAQV-REQUIMTE, Department of Chemistry, NOVA School of Science and Technology, FCT NOVA, Universidade NOVA de Lisboa, 2829-516 Caparica, Portugal; jgc@fct.unl.pt; 5 Instituto de Tecnologia Química e Biológica António Xavier, Universidade Nova de Lisboa, Av. da República, 2780-157 Oeiras, Portugal

**Keywords:** membrane stability, UV photodegradation, TiO_2_ coating, hybrid reactor, hormone removal

## Abstract

The combination of photocatalysis and membrane filtration in a single reactor has been proposed, since the photocatalytic treatment may degrade the pollutants retained by the membrane and reduce fouling. However, polymeric membranes can be susceptible to degradation by UV radiation and free radicals. In the present study, five commercial polymeric membranes were exposed to ultraviolet (UV) radiation before and after applying a sol–gel coating with TiO_2_ nanoparticles. Membrane stability was characterized by changes in hydrophilicity as well as analysis of soluble substances and nanoparticles detached into the aqueous medium, and by Fourier transform infrared spectroscopy (FTIR), scanning electron microscope (SEM), and energy-dispersive X-ray spectrometry (EDS) for structural, morphological, and elemental distribution analysis, respectively. The TiO_2_ coating conferred photocatalytic properties to the membranes and protected them during 6 h of UV radiation exposures, reducing or eliminating chemical and morphological changes, and in some cases, improving their mechanical resistance. A selected commercial nanofiltration membrane was coated with TiO_2_ and used in a hybrid reactor with a low-pressure UV lamp, promoting photocatalysis coupled with cross-flow filtration in order to remove 17α-ethinylestradiol spiked into an aqueous matrix, achieving an efficiency close to 100% after 180 min of combined filtration and photocatalysis, and almost 80% after 90 min.

## 1. Introduction

Polymeric materials are the first choice for large-scale membrane separation processes due to the variety of structures and properties of polymers, which allows the production of membranes with diverse characteristics, covering an extensive range of molecular weight cut-offs (MWCO), with a low cost of production in comparison with ceramic membranes [1,2,3,4].

Coupling membrane filtration with photocatalytic processes may minimize chemical and biological membrane fouling due to the degradation of organic substances present in the feed/retentate by UV radiation [4,5]. However, the degradation of polymeric membranes by UV radiation is an important limitation to their use in hybrid systems.

TiO_2_ nanoparticles are effective photocatalysts reported as low cost, attractive materials for water treatment and have been tested and associated with the membrane filtration processes [6]. The TiO_2_ nanoparticles can be incorporated into polymers or used to coat membrane surfaces to produce composites and hybrid materials [3,4,5,6,7,8,9]. Different approaches have been used to modify the surfaces of polymeric membranes with TiO_2_ [7,8,9,10,11,12]. Pre-coating approaches promote the functionalization of polymeric membranes’ surfaces, allowing the necessary chemical conditions to the inorganic TiO_2_ fixation. These approaches have been applied successfully to modify common membranes such as polyamides, polyethersulfone (PES), polypropylene (PP), cellulose acetate (CA), polyvinylidene fluoride (PVDF), and others [7,8]. Previous studies have shown that UV exposure can increase the hydrophilicity of the membrane, which might improve the filtration processes [13]. However, other studies reported the negative effect of UV radiation on membranes [14,15,16,17,18].

Two critical factors to consider are the effect of exposure of membranes to UV radiation and the effect of the hydroxyl radicals (^●^OH) produced by the photocatalyst TiO_2_ under UV radiation, which can severely damage the polymeric structure [18,19,20]. In addition, there is strong evidence that smaller plastic particles can be released from polymers exposed to UV radiation (90% predominance of particles smaller than 200 nm), and these particles can present a higher dramatic negative impact on biota than larger ones [21,22].

Since the chemical structure of the polymer will influence its susceptibility to UV exposure, the evidence of resistance of some polymers can guide the choice and development of membranes to be used and further tested in combination with UV radiation. These concerns justify the need to assess the stability of polymeric membranes to UV radiation before they may be proposed for water and wastewater treatment combined with photodegradation and photocatalysis.

In the present work, five commercial polymeric membranes made of polymeric materials commonly used for microfiltration, nanofiltration, or reverse osmosis were chosen to evaluate their stability against exposure to UV radiation employing a mercury medium pressure UV lamp that emits polychromatic light at a wide diversity of wavelengths. In addition, the membranes were also modified by coating with TiO_2_ nanoparticles employing a sol–gel coating method. The photocatalytic activity of modified membranes and the effect of the coating layer in protecting the integrity of membranes were investigated. The main goal was to verify if the coating with TiO_2_ nanoparticles would provide protection to the membranes or contribute to their degradation by the production of the highly reactive ^●^OH on their surfaces. The effect of UV exposure on the selected membranes was monitored by measuring the detachment of chemical compounds and nanoparticles into the water matrix used in exposure experiments and by checking morphological, chemical, and hydrophobicity changes in the membranes. In addition, modified and unmodified nanofiltration membranes were evaluated in a hybrid reactor to test if they could be used to retain and degrade 17α-ethinylestradiol, which has been widely reported to occur in the aquatic environment [23,24].

## 2. Experimental Section

### 2.1. Materials and Methods

#### 2.1.1. Prepare of Photocatalytic Membranes

**Figure 1:**
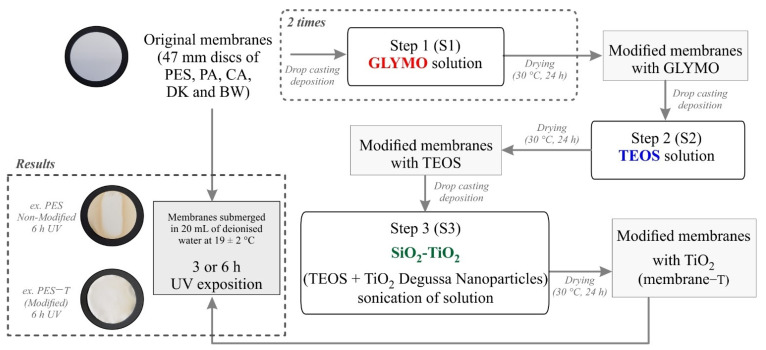
Flow chart depicting the sol–gel procedure followed to coat polymeric membranes with TiO_2_ and the UV exposure assays conducted to evaluate the membranes’ stability.

Five commercial polymeric membranes used for microfiltration, nanofiltration, and reverse osmosis were tested in this study: Polyethersulfone 0.2 µm (PES) (GelmanSciences, Ann Arbor, MI, USA), Polyamide-Nylon 0.2 µm (NYLON) (Whatman, Maidstone, London, UK), Cellulose Acetate 0.45 µm (CA) (Whatman, Tokyo, Japan), the Polyamide Thin-Film Composite membranes DK (GE, Trevose, PA, USA), as well as BW30-400 (BW) (Dupont, FilmtecTM Membranes, Miami, FL, USA). The chosen membranes are composed by different polymeric materials and have different pore diameters. Information about the structural properties of the commercial membranes provided by the manufacturers is available as Supplementary Data (Table S1). Discs with 47 mm diameter of each membrane were thoroughly washed with deionized water, ultrasonicated for 10 min to remove any contaminants, and dried overnight (24 h, 30 °C). The photocatalytic membranes were prepared following an adapted sol–gel methodology previously proposed [25,26]. The sol–gel coating was conducted by sequential steps (denoted as S) using 2 mL drop-casting solutions: (S1) GLYMO coating, (S2) TEOS coating, and (S3) SiO_2_-TiO_2_ coating. Each drop-casting step was followed by a drying step of 24 h at 30 °C (Figure 1). Details about the modification procedure are provided in the Supplementary Data.

After each layer coating, the membranes surface chemical composition was analyzed by Fourier transform infrared spectroscopy (FTIR). The modified and original membranes were exposed to UV radiation to evaluate their stability after radiation, as detailed in Section 2.1.3. Figure 1 shows a general scheme of the modification and UV exposure procedures.

#### 2.1.2. Evaluation of the Photocatalytic Activity of the Modified Membranes

The photocatalytic activity of the modified membranes was evaluated using the method described by Elovitz (1999) [27], which employs *p*-chlorobenzoic acid (*p*CBA, 99%, Aldrich, Germany) as a hydroxyl radical (^●^OH) probe compound. The choice of *p*CBA is based on its ready reaction with ^●^OH, *k_OH/pCBA_* = 5 × 10^9^ M^−1^ s^−1^, which could be generated on the surfaces of the modified photocatalytic membranes exposed to UV radiation [7,8]. Disks with 47 mm diameter of the unmodified and modified membranes were individually immersed in 20 mL of a 500 µg/L *p*CBA solution placed in stirred refrigerated double-walled glass Petri dishes kept at 18 ± 2 °C. The samples were placed inside a collimated beam reactor and exposed for 1 h to UV radiation employing a medium pressure mercury lamp UVH-Lamp Type Z (UV-Technik, Luton, UK) placed 17 cm above the solution. Dark controls (without UV exposure) were also tested. Samples, aliquots of 1 mL of the *p*CBA solutions, were collected before and after the dark and UV exposure experiments. The *p*CBA concentration was determined by injecting 70 µL to be analyzed by High-Performance Liquid Chromatography (HPLC) using a Water system (Alliance e2695 Separations Module, LabX, Midland, ON, Canada) equipped with a photodiode array detector (PAD, 2998, Waters Corporation, Milford, MA, USA). The pCBA signals were separated using a Luna 5 µ C18(2) 100A (150 × 3.0 mm) column (Phenomenex Inc., Torrance, CA, USA) kept at 40 °C and applying an isocratic method (mobile phase: 50% acetonitrile + 50% of water with 0.1% of formic acid) with a flow rate of 0.6 mL/min. The *p*CBA detection was performed at λ = 238 nm, and the analyte concentrations were determined based on calibration curves, with a direct-injection detection limit of 10 µg/L. The efficiency removal of *p*CBA was calculated using the following equation (Equation (1)):

(1) $$\%\;\mathrm{Efficiency}\;\mathrm{removal}=\frac{C_{0}-C_{t}}{C_{0}}\times 100$$

where *C*_0_ is the initial average concentration and *C_t_* is the average concentration of the compounds at the end of the degradation procedures.

#### 2.1.3. Exposure of Non-Modified and Modified Membranes to UV Radiation

The unmodified and modified membranes were exposed to UV radiation to assess the damaging effects of radiation on the polymeric structure. For this purpose, discs with 47 mm diameter of the unmodified membranes (PES, CA, NYLON, DK and BW) were placed in a temperature-controlled (18 ± 2 °C) double-walled glass Petri dish, containing 20 mL of previously autoclaved and filtered (0.2 µm) deionized water. This setup was submitted to 3 or 6 h of UV exposure employing the same reactor mentioned in Section 2.1.2. After exposure, the aqueous samples were analyzed using the techniques described below. Dark controls (not subject to UV exposure) were also tested. The same experiment was conducted for 6 h for all membranes modified by the sol–gel with TiO_2_ (labeled as PES-T, CA-T, NYLON-T, DK-T, and BW30-T). The well-described PES degradation by UV [28,29] served as basis to study the potential protective influence of each covering layer (described in Section 2.1.1) in the modified PES membranes. This experiment was conducted during 3 h of UV exposure and performed in quadruplicate.

After UV exposure, the aqueous media were collected, and the volumes were completed up to 25 mL with autoclaved and filtered (membrane disk of 0.2 µm pore size) deionized water. The water samples were analyzed by UV-Vis spectrometry in scan mode (Ultrospec 2010 pro UV-Vis, Amersham Biosciences, Amersham, UK) and Nanoparticle Tracking Analysis (NTA, NanoSight NS300 Malvern with a laser of 405 nm, Malvern Panalytical, Malvern, UK) to verify, respectively, the possible presence of soluble substances and detached particles from the membranes in the aqueous samples after UV exposure. The NTA detects particles in the 30–2000 nm size range. The detection of nanoparticles in the samples was performed by three video runs of 30 s with 749 frames/s employing a blue 488 laser, an sCMOS camera, a slider 1259 shutter, and a slider gain of 366 as capture settings. The captured images were processed by NTA 3.3. software, whereas the DevBuilt 3.3.301 software (Malvern Panalytical, 2018, Almelo, Netherlands) provided information on the particle size distribution and their concentration.

The membrane discs were dried in an oven (24 h at 30 °C), photographed, and preserved in a desiccator until characterization by FTIR and Scanning Electron Microscopy (SEM), as detailed in Section 2.2.

### 2.2. Characterization of Membranes before and after UV Radiation Exposure

The morphology (top and cross-section) of the unmodified and modified membranes, before and after UV exposure, were analyzed by scanning electron microscopy (SEM) using various magnifications and energy-dispersive X-ray spectrometry (EDS) mapping analysis to check the elemental composition of the membranes. The membrane’s thickness before and after TIO_2_ coating was measured using a MDC-25SX Digimatic Micrometer (Mitutoyo, Kanagawa, Japan) in at least three different random places. The chemical structure of the unmodified and modified membranes before and after UV radiation exposure was analyzed by Fourier transform infrared spectroscopy (FTIR) using attenuated total reflectance (ATR) mode.

The hydrophilicity of the unmodified and modified membranes before and after UV exposure was evaluated by measuring the water contact angle by the sessile drop method using A KSV CAM2008 equipment [30]. Further descriptions of analytical conditions, measurement experiments, and equipment are available as Supplementary Data.

### 2.3. Membrane Filtration Assays for Hormone Removal in Water

From the results obtained in terms of chemical resistance, photoactivity, and due to their suitable molecular weight cut-off, DK and DK-T membranes were selected for further testing to address the potential of the membranes to remove chemical pollutants from water using a treatment process that combines nanofiltration and photolysis in a single reactor. DK and DK-T membranes were used to remove 17α-ethinylestradiol from water employing a hybrid reactor previously used and described by Oliveira et al. (2020) [31]. An image of the reactor is available in the Supplementary Data (Figure S1). A low-pressure mercury UV lamp (OSRAM HNS G5 6W UVC Germicidal PURITEC lamp, G6T5/OF RG3, Osram, Munich, Germany) that emits monochromatic light at 254 nm was used in the studies of photolysis and photocatalysis. The distance between the lamp and the membrane was maintained at 9.0 cm, and the surface area available to be radiated by the UV lamp was 36 cm^2^. Seven different assays were conducted to elucidate the phenomena that could be involved in hormone removal from the water solution: (a) Evaluation of hormone adsorption on the surface of the reactor components; (b) Direct photolysis without membrane, (c) Direct photolysis and photocatalysis with modified DK-T membrane, without filtration; (d) Filtration with unmodified DK membrane; (e) Filtration with modified DK-T membrane, (f) Photolysis associated to filtration with unmodified DK membrane, and (g) Photocatalysis associated to filtration with modified DK-T membrane. All assays were conducted in full recirculation mode during 3 h. T_0_ was the aliquot collected after 15 min of recirculation, to determine the initial hormone concentration in the working solution, which was probably diluted because of the aqueous mixture hormone solution used for the conditioning of the filtration membranes.

It should be noted that in studies employing UV radiation, the lamp was switched on and stabilized for 20 min before the exposure of the solution to radiation and, until the moment the first aliquot was collected, the shutter was closed. The following collections were taken every 30 min, with 2 mL samples taken from the feeding bottle, which contained 500 mL of a 500 µg/L solution of 17α-ethinylestradiol prepared with deionized water. The solutions were prepared daily by appropriate dilution of a stock solution of 17α-ethinylestradiol previously dissolved in acetonitrile (Sigma-Aldrich, Hamburg, Germany) and the feed bottle was maintained temperature-controlled at 18 ± 2 °C. The collected samples and work solutions were filtered (0.2 µm) and frozen at −20 °C until analysis by high-performance liquid chromatography (HPLC). A 6 min isocratic HPLC method (40:65 acetonitrile/water) was used with a flow rate of 1 mL/min, oven temperature of 30 °C, 50 μL of sample injection, and employing a λ = 250 nm (direct injection detection limit of 50 μg/L). The HPLC equipment used was described in Section 2.1.2.

## 3. Results and Discussion

### 3.1. Evaluation of the Photocatalytic Activity of the Modified Membranes

All the modified membranes exposed to UV presented the efficiency of *p*CBA degradation among 61.6 to 96.0%, except the PES-T that degraded around 12 ± 1% (Figure S2, Supplementary Data). These results indicate that modification with the TiO_2_ layer displayed different photocatalytic activities depending on the material of the modified membranes. Considerations regarding the low degradation efficiency of the PES-T membrane are discussed in Section 3.2.

### 3.2. Exposure of Non-Modified and Modified Membranes to UV Radiation

The exposure to UV radiation might promote damages in the polymeric structure, being mostly remarkable in those polymers portraying chromophores’ groups, producing very reactive free radicals by photolysis [20,32,33], which induce fragmentation photooxidation mechanisms and alter their chemical composition. Thus, those polymers are more prompt to change their characteristics such as color or mechanical resistance and, consequently, their functionalities, which is especially relevant to consider if used as membranes in water treatment processes. The degradation process of polymeric materials can produce soluble substances that are potentially toxic and detach to water small harmful polymeric particles, which are classified as microplastics and nanoplastics pollutants [21,34].

#### 3.2.1. Visual Evaluation and Resistance to Manipulation of Non-Modified Membranes after UV Radiation

All unmodified membranes remained resistant to manipulation after UV exposure, except for the CA membrane that became curved, very fragile, and vulnerable to manipulation (Figure S3, Supplementary Data), suggesting an important negative UV effect over this membrane. This result is in accordance with previous studies that tested similar cellulose acetate-based materials and reported their photochemical degradation by UV radiation [35].

A visual evaluation of unmodified PES, DK, and BW membranes showed that the color of their surfaces was dramatically affected by UV exposure (Figure S3, Supplementary Data), also showing a non-uniform light yellowish-brown appearance on their surface with light and dark zones. The appearance of different colored zones could be related to the collimated beam tests made under UV light above the surface of the reactor (Figure S4, Supplementary Data). Despite assuming that the light beams are parallel to each other and perpendicular to the irradiated surface, the complexity of the UV distribution in the reactor leads to non-uniform irradiation due to the reflectance on its surfaces [36].

Since PES is an example of a photo-instable polymer [33], it was used to evaluate the efficiency of the sol–gel coating protection. PES comprises a chromophore group, the phenoxy-phenyl sulfone group, which is rich in π bonds that can interact with UV radiation, degrading and yellowing when PES is exposed to UV light [28,37]. Before and after each layer deposition, the PES membranes were exposed to 3 h of UV radiation. The pictures of membranes covered with each layer before and after UV exposure are presented in Figure S5 (Supplementary Data). The images show that the unmodified PES membrane was damaged by UV radiation as expected [38], as well as the membranes PES-G (2 GLYMO layers) and PES-TEOS (2 GLYMO layers + 1 TEOS layer), which presented light and dark zones. Thus, the presence of SiO_2_ provided by GLYMO and TEOS precursors was not enough to prevent UV damage completely. Dark zones appeared in the regions where the UV radiation was more intense due to the reflection of the light emitted by the lamp by metallic shields that make up the reactor. On the contrary, for the PES-T-modified membrane, there was not any observed darkening of the membranes, suggesting a higher degree of UV protection provided by the TiO_2_ layer.

#### 3.2.2. UV-Vis Spectroscopy to Monitor the Influence of the Coating Layers as Protection to UV Radiation Exposure

**Figure 2:**
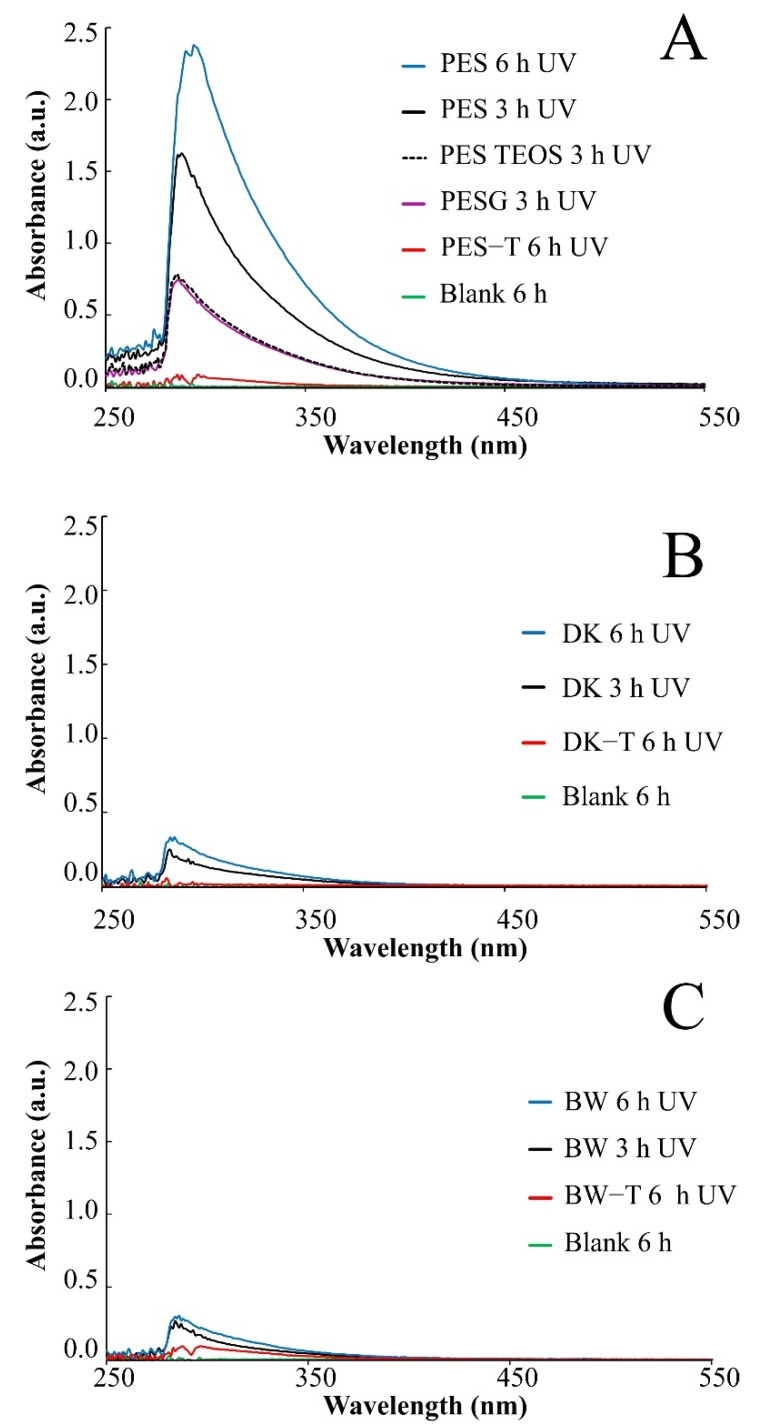
UV-Vis spectra for aqueous samples recovered after UV exposure of the membranes. (**A**) PES: Polyethersulfone (0.2 µm) before and after each coating layer of sol–gel modification (PES-G, PES-TEOS, and PES-T); (**B**) DK and (**C**) BW before and after sol–gel modification with TiO_2_ (DK-T and BW-T).

Color changes were observed in the aqueous media collected for the PES unmodified membrane exposed to UV radiation (3 and 6 h), denoting a release of soluble substances that turned the aqueous solution yellow (Figure S6, Supplementary Data), which was monitored by UV-Vis spectroscopy (Figure 2), suggesting degradation of the polymer.

Nevertheless, for modified PES-G, PES-TEOS, and modified PES-T, DK-T, and BW-T membranes, the UV-Vis spectra analyses showed a lower intensity in the absorbance band in the same region (Figure 2A–C), indicating a lower concentration of soluble substances released to the aqueous media. The decrease in the color intensity in the UV-Vis spectra of the aqueous media, collected after UV exposure observed for PES-G and PES-TEOS membranes, after UV irradiation (Figure 2A), reinforces the hypothesis that both layers also contribute to protecting the membranes from UV degradation in some way, which is in accordance with pictures of PES membranes unmodified and covered with each layer before and after exposure (Figure S5, Supplementary Data). Similarly, the absence of UV absorbance signals observed for PES-T, DK-T, and BW-T suggest that the TiO_2_ layer protects very effectively the membranes from UV radiation during the exposure times tested.

Neither color nor absorbance signals were observed in the aqueous media when CA and NYLON membranes were exposed to the same conditions of UV radiation, suggesting that these membranes did not release soluble substances to the aqueous matrix. However, the degradation of CA was evident not only due to the change of its appearance but also its resistance to manipulation after being irradiated (Figure S3, Supplementary Data).

#### 3.2.3. SEM Analysis

**Figure 3:**
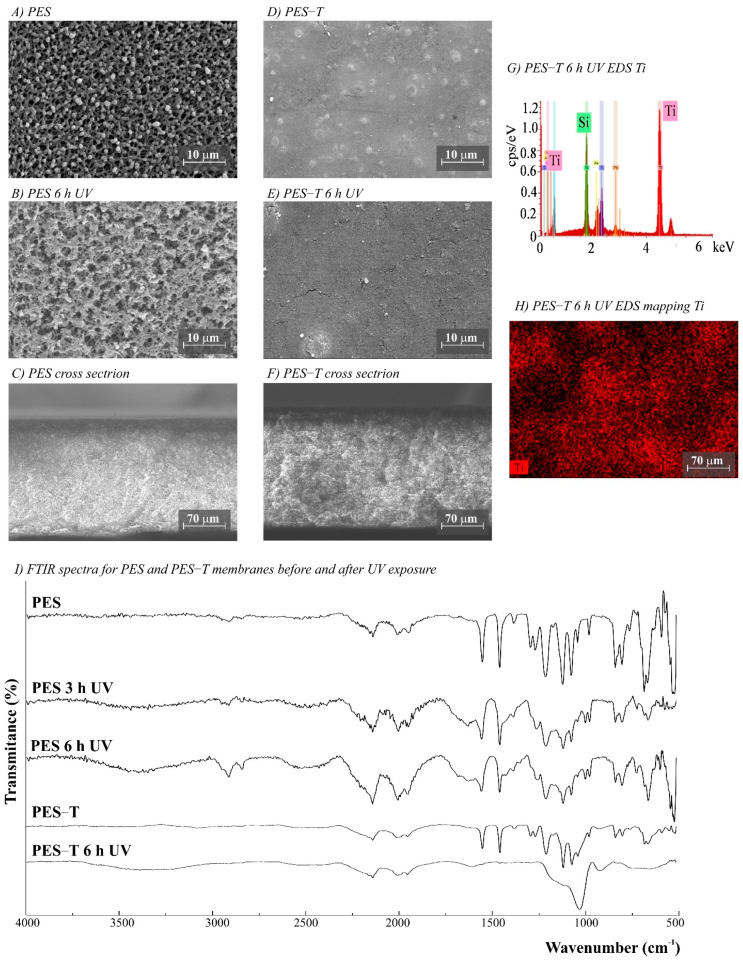
(**A**–**F**) SEM images for PES (polyethersulfone, 0.2 µm) and PES-T (PES modified with TiO_2_) membranes before and after UV exposure (magnifications ×3000), (**G**) PES-T EDS, (**H**) EDS mapping showing the Ti distribution on the membrane surface after 6 h UV exposure, and (**I**) Fourier transform infrared spectroscopy (FTIR) spectra for PES and PES-T membranes before and after 3 and 6 h of UV radiation.

**Figure 4:**
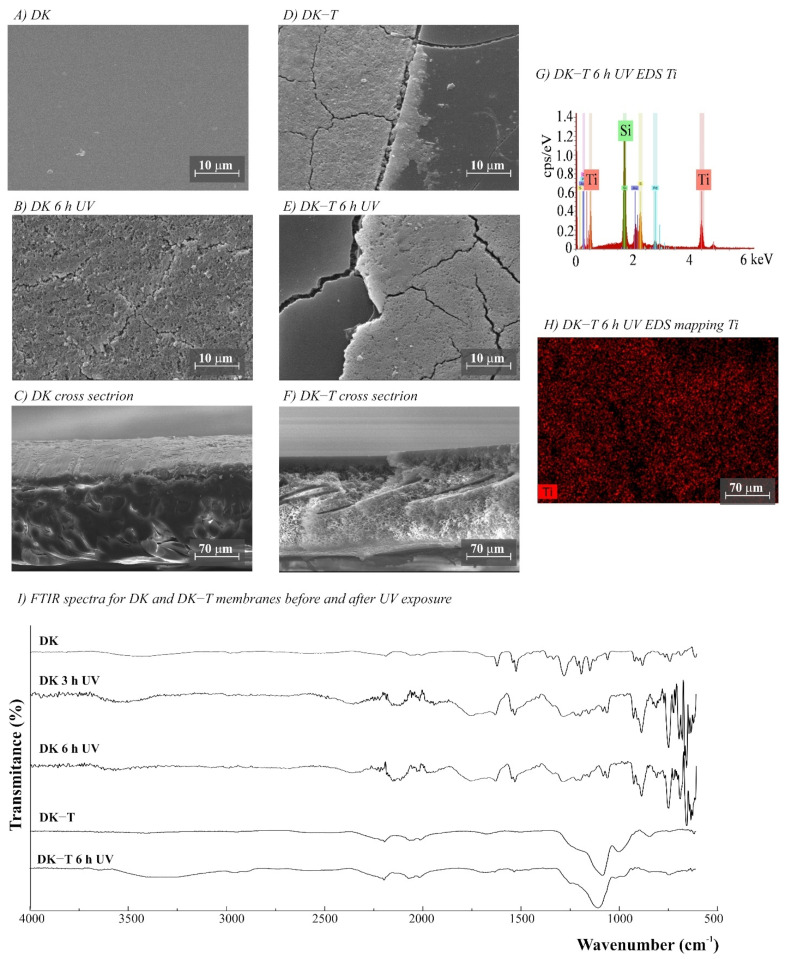
(**A**–**F**) SEM images for DK and DK-T (DK modified with TiO_2_) membranes before and after UV exposure (magnification ×3000), (**G**) DK-T EDS, (**H**) EDS mapping showing the Ti distribution on the membrane surface after 6 h UV exposure, and (**I**) Fourier transform infrared spectroscopy (FTIR) spectra for DK and DK-T membranes before and after 3 and 6 h of UV radiation.

**Figure 5:**
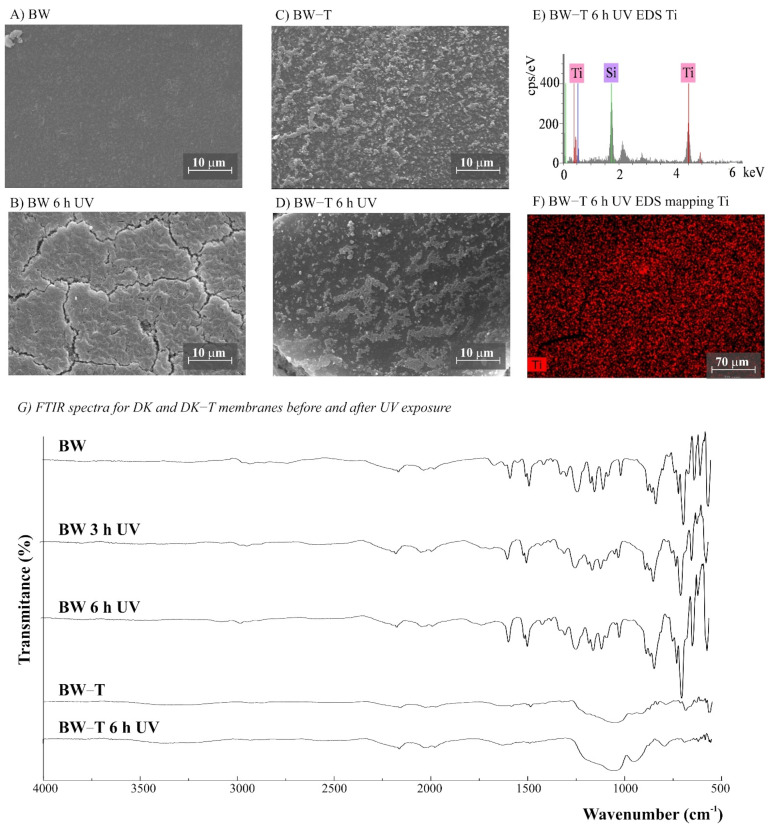
(**A**–**D**) SEM images for BW and BW-T (BW modified with TiO_2_) membranes before and after UV exposure (magnification ×3000), (**E**) BW-T EDS, (**F**) EDS mapping showing the Ti distribution on the membrane surface after 6 h UV exposure, and (**G**) Fourier transform infrared spectroscopy (FTIR) spectra for BW and BW-T membranes before and after 3 and 6 h of UV radiation.

**Figure 6:**
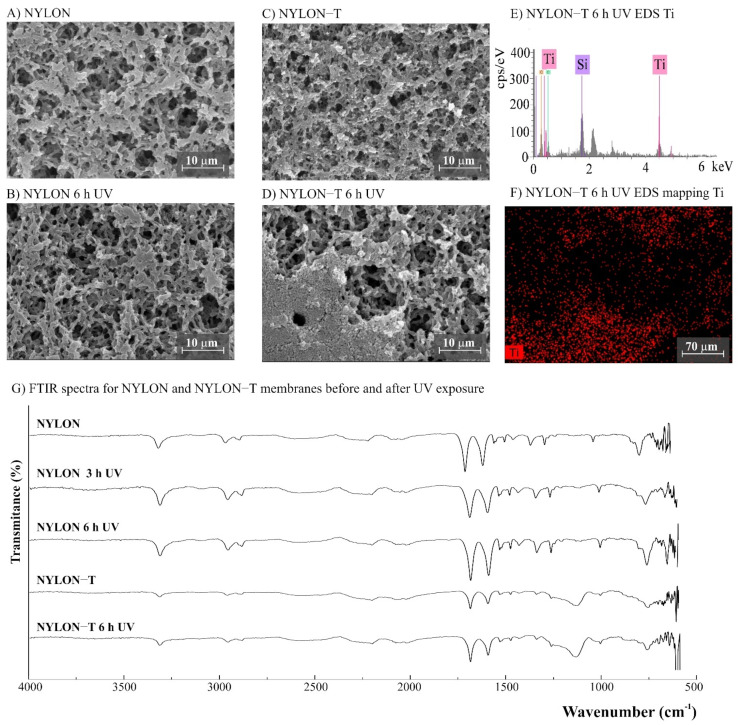
(**A**–**D**) SEM images for NYLON (polyamide–nylon, 0.45 µm) and NYLON-T (NYLON modified with TiO_2_) membranes before and after UV exposure (magnification ×3000), (**E**) NYLON-T EDS, (**F**) EDS mapping showing the Ti distribution on the membrane surface after 6 h of UV exposure, and (**G**) Fourier transform infrared spectroscopy (FTIR) spectra for NYLON and NYLON-T membranes before and after 3 and 6 h of UV radiation.

**Figure 7:**
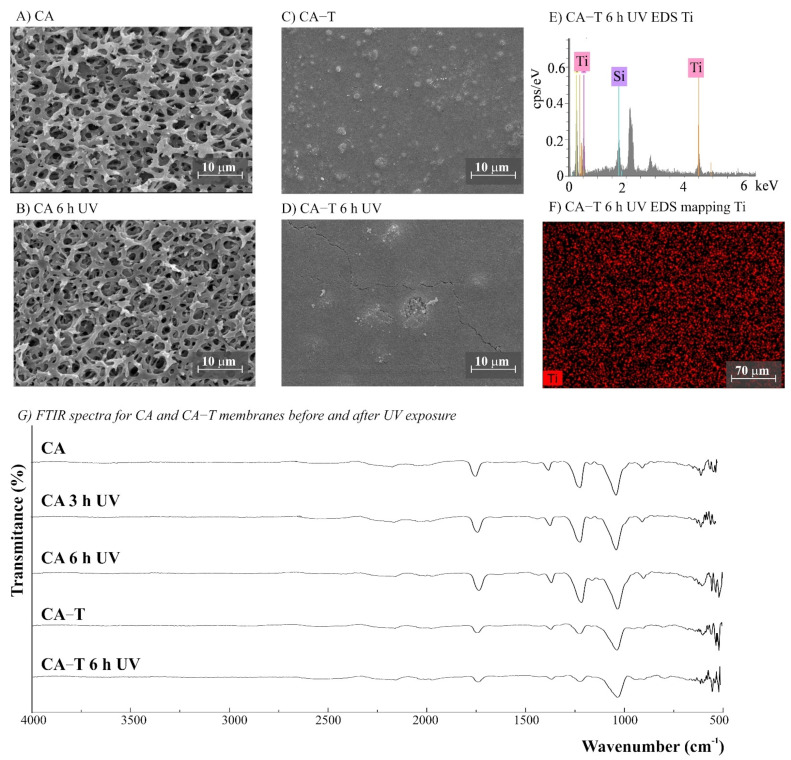
(**A**–**D**) SEM images for CA (cellulose acetate) and CA-T (CA modified with TiO_2_) membranes before and after UV exposure (magnification ×3000), (**E**) CA-T EDS, (**F**) EDS mapping showing the Ti distribution on the membrane surface after 6 h UV exposure, and (**G**) Fourier transform infrared spectroscopy (FTIR) spectra for CA and CA-T membranes before and after 3 and 6 h of UV radiation.

The morphology of the membranes was analyzed before and after UV radiation exposure (Figure 3, Figure 4, Figure 5, Figure 6 and Figure 7). It is possible to observe changes promoted by the TiO_2_ coating in all modified membranes, with a more homogeneous coating for the membranes with larger porous size, which is observed both by SEM images and Ti atom distribution mappings obtained for TiO_2_-modified membranes exposed for 6 h to UV radiation. Different aspects could influence the quality of the membranes’ coating shown in SEM, such as the morphology and nature of the substrate, the crystalline phase used, or the packaging of the photocatalytic particles on the surface of the substrate [39,40]. Rough and porous substrates generally show more uniform deposition and coatings due to better adhesion of TiO_2_ particles, and this could lead to improved photocatalytic activity [39,40]. This could explain the different homogeneity of the modified membranes. For example, the DK-T and BW-T membranes exhibited low homogeneity with some cracks after the TiO_2_ coat (Figure 4D and Figure 5D) compared to the membranes PES-T (Figure 3D), NYLON-T (Figure 6C), and CA-T (Figure 7C).

Comparison of the cross-section images for the membranes PES, PES-T, DK, and DK-T are also presented (Figure 3C,F and Figure 4C,F), showing that the sol–gel layers deposited at the surface did not penetrate the polymeric substrate. Moreover, for the DK-T membrane (Figure 4D,F), it was possible to observe a layer with crystalline-dense characteristics attributed to the previous deposition of the TEOS layer on the surface of the membranes, which (as described above) was not homogeneously covered by the TiO_2_ layer. In any case, the TEOS layer seems to provide some protection against UV radiation, as discussed previously in the study of the influence of the coating layers on protection to UV radiation.

For NYLON and CA membranes, it was possible to observe that the exposure to UV radiation for NYLON and CA membranes causes less accentuated damage to their surfaces (Figure 6B and Figure 7B), compared with their non-irradiated membranes (Figure 6A and Figure 7A), suggesting that the polymers of these membranes are less damaged. However, after UV exposure for the CA-T membrane (Figure 7C,D), it is possible to observe that the coat maintained its homogenous integrity compared to the unmodified CA membrane (Figure 7A,B), denoting an improvement in its chemical–mechanical manipulation resistance.

Probably, the morphological preservation observed for the coated membranes exposed to UV radiation was a protection effect promoted by the presence of Ti, which was homogeneously distributed on the membrane surfaces by the proposed sol–gel modification method, as shown in the EDS maps (Figure 3H, Figure 4H, Figure 5F, Figure 6F and Figure 7F). The thicknesses for all original and modified membranes were measured in triplicate and they were not statistically significant (Table S2).

#### 3.2.4. Comparison of FTIR Spectroscopy Analysis for Membranes before and after Modification and UV Exposure

The FTIR spectra for unmodified and modified PES, CA, NYLON, DK, and BW membranes are shown in Figure 3, Figure 4, Figure 5, Figure 6 and Figure 7. Additionally, the FTIR spectra of the commercial PES membrane before and after each modification coating (exposed or not to UV radiation) are presented in Figure S7 (Supplementary Data). Comparing the unmodified membrane with the membrane covered with the SiO_2_ precursor GLYMO, it was possible to observe changes in the region between 1050 and 1000 cm^−1^ associated with Si–O–Si, Si–O–C, Si–O–H, and C–O bands and at 910 cm^−1^ (oxirane group from the GLYMO structure) in the modified membrane, depicting the formation of a hybrid SiO_2_ structure at the PES membrane surface, while the main peaks for the commercial PES [41] were maintained. The Si–O groups formed from the crosslinker GLYMO were necessary to make the organic polymeric surface compatible with inorganic structures such as SiO_2_, which was formed after hydrolysis of the TEOS precursor used. The differences observed for bands in the Si-O region between 1072 and 975 cm^−1^ for the PES-TEOS membrane and the disappearance of the 910 cm^−1^ bands for oxirane groups confirmed the fixation and compatibilization of silica at the polymeric membrane surface. The spectrum for PES-T modified with SiO_2_-TiO_2_ (Figure 3I) showed some differences in the Si–O region, 1100–750 cm^−1^, which corresponds to the stretching Si–O–Ti vibration [42] and, more specifically, the peak at 950 cm^−1^ [35] endorsing the presence of the TiO_2_ photocatalyst on the membrane surface.

For the membranes DK, DK-T, BW, and BW-T membranes (Figure 4L and Figure 5G), the changes promoted by TiO_2_ coating were much more evident. Therefore, the FTIR analysis made for the PES-T modified membrane is also applicable to DK-T and BW-T, with the Si-O bands (1130 and 987 cm^−1^) and the Si–O–T stretching band (950 cm^−1^). However, the DK-T and BW-T spectra showed a very similar profile to the commercial Degussa P25 TiO_2_ powder FTIR spectrum [43], with the peak associated with Ti–O–Si at around 950 cm^−1^ [33] and Si-O bands (1260–700cm^−1^).

The FTIR spectra for NYLON-T and CA-T membranes showed that the main chemical structure remained unchanged compared to the unmodified CA and NYLON membranes, presenting discreet changes at 1100–750 cm^−1^, assigned to stretching Si-O-Ti vibration (Figure 6G and 7G). Moreover, similarly to the modified PES-T membrane, they showed slight changes in the region between 1100 and 750 cm^−1^.

For the membranes exposed to UV radiation, the visual changes on the membrane surfaces (Figure S3, Supplementary Data) can be linked with the considerable differences observed in the normalized FTIR spectra between the unmodified PES membrane before and after 3 and 6 h of UV exposure (Figure 3I). Additionally, there were similarities between the membranes exposed for 3 and 6 h. The chemical structure of the PES polymer comprises three main chemical functional groups; all are observed in the spectra presented in Figure 3I: aromatic rings (around 1600 cm^−1^), ether (around 1400–1300 cm^−1^), and sulfone (1200 to 1100 cm^−1^) [44]. However, after UV exposure, it was possible to observe important changes in the PES spectrum. The appearance of typical C=C alkenes stretching (1800 to 1640 cm^−1^) and the loss of resolution of the peaks of aromatic rings suggest a conversion from an aromatic to an aliphatic structure. Additionally, there is a loss of peak definition in the region between 1340 and 900 cm^−1^, suggesting changes in the polymer’s ether and sulfone functional groups.

The DK and BW membranes released soluble substances detected by UV-Vis in the aqueous solutions collected after UV exposure (Figure 2B,C, respectively). These membranes also presented regions with different colors on their surfaces (Figure S3, Supplementary Data) and significant changes in FTIR spectra after exposure to UV radiation (Figure 4I and Figure 5G, respectively). The FTIR spectra for these two polyamide membranes show that DK and BW are much more similar to each other than with NYLON, with the difference that BW is a polyamide thin film composite (TFC) and DK is a skin layer polyamide, coated with a hydrophilic neutral layer rich in –OH groups, with peaks between 3000 and 2700 cm^−1^ [45,46,47]. The commercial polyamide TFC and skin layer membranes are typically composed of three layers: a supported web of polyester, a polysulfone porous mid-layer, and a full aromatic polyamide cover layer [48]. As both DK and BW membranes presented a loss of peaks resolution in bands assignable to polysulfone (between 1420 and 990 cm^−1^) after UV exposure, the polysulfone mid-layer was probably susceptible to degradation, despite the polyamide layer. This result corroborates and explains the UV-Vis spectra obtained for released soluble substances to the aqueous medium for DK and BW30 membranes that absorbed in the same region observed for soluble substances released by the PES membrane.

No chemical changes were observed for FTIR spectra of unmodified NYLON and CA membranes before and after UV exposure (Figure 6G and Figure 7G), which corroborates the results obtained by aqueous samples. However, photodegradation can degrade the cellulose acetate, and it is reported to be induced by the formation of free radicals [49]. Studies involving the irradiation of CA fiber with UV light denoted two degradation mechanisms: cleavage of side groups and polymer chain scission of the glucoside bond. Four different radicals were identified due to the cleavage of the lateral acetate groups, the glucoside bonds, and the abstraction of hydrogen [50]. The effect of UV radiation on CA membranes could reduce dramatically its molecular weight and affect their resistance [51]. Although chemical changes were not evidenced by the FTIR spectra (Figure 7G), the CA membranes exposed to UV radiation became brittle, losing their resistance to manipulation (Figure S3). For CA-T, the modification conferred mechanical resistance to the membrane, which was probably due to the contribution of the silicon layers [31,41]. Similar to original membranes, there were no differences observed in the FTIR spectra for both CA-T and NYLON-T membranes before and after UV.

The NYLON polymeric structure consists of a saturated aliphatic chair without π bonds between carbon atoms in its chemical structures, which makes it less susceptible to the absorption of ultraviolet (UV) radiation [30]. This characteristic can explain the stability of the polymeric structure and the mechanical stability of the membranes after UV radiation exposure.

#### 3.2.5. NTA Analysis

The NTA analysis does not determine the chemical nature and composition of the particles released, but it is helpful to monitor, for example, the potential presence of nanoplastic and microplastic potentially detached from membranes promoted by UV radiation. This is a serious concern due to the toxic effects of nanoplastics related to their bioaccumulation and the transport of hazardous substances and pathogenic microorganisms, which have already started to be investigated [52]. Additionally, the actual potable water treatment processes do not comprise operations that assure the removal of nanoplastics.

**Figure 8:**
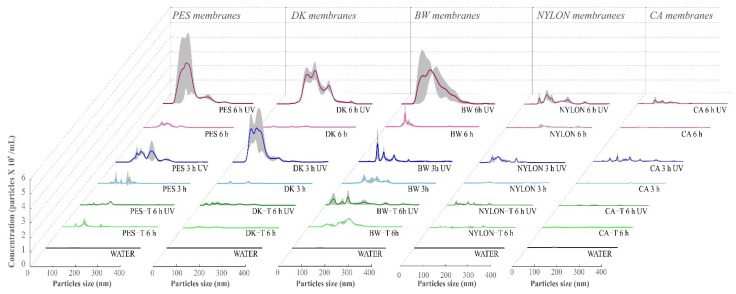
Nanoparticle tracking analysis (NTA) of the aqueous media (WATER) in contact with the unmodified and modified membranes after 3 or 6 h exposure to UV radiation. The gray regions show the variation of results obtained in four replicate experiments. Non-modified membranes: Polyethersulfone (PES, 0.2 µm), Cellulose Acetate (CA, 0.45 µm), Polyamide–Nylon (NYLON, 0.45 µm), DK and BW; and modified membranes: PES-T, CA-T, NYLON-T, DK-T, and BW-T.

Observing the particle size distribution and their concentrations (Figure 8, and Table 1), there is an evident influence of UV radiation exposure on the detachment of solid particles from non-modified membranes to the aqueous media. The increase in the UV exposure time amplified the concentration and the diversity of the size of the detached particles detected by NTA. Particle detachment was more relevant for the PES, DK, and BW unmodified membranes (Table 1).

For all the unmodified membranes except CA, the detachment of particles to the aqueous media was more significant after 6 h of UV exposure than after 3 h (3.2, 1.3, 9.5, 0.8, and 1.9 times higher for PES, DK, BW, CA, and NYLON, respectively). However, among them, CA and NYLON membranes detached considerably fewer particles to the aqueous media, presenting the same order of magnitude as the values obtained for dark controls (membranes that were not exposed to UV radiation).

Considering the NTA analyses (Figure 8, and Table 1), the coating with TiO_2_ nanoparticles considerably reduces the detachment of solid particles from membranes to the aqueous media promoted by the UV radiation exposure. The concentration of particles observed for membranes coated with TiO_2_ was similar to the dark controls, where 90% of the particles detected present a size lower than 150 nm, suggesting effective protection promoted by the membrane coating with TiO_2_, despite the free radicals formed at the membrane surface in the photocatalytic process.

The particles observed for control samples (dark controls without UV exposure) probably represent the naturally occurring particles that were introduced into the samples from different experimental sources (e.g., atmospheric dust that can be deposited during the experiment or dragged into the aqueous medium during bottle transfer and aliquot procedures and remains of particles on the surface of the flasks used in the experiments and storage). An illustrative NTA video of DK and DK-T membranes samples is available as Supplementary Data (Video S1).

The NTA results corroborate the SEM and FTIR analyses, showing that degradation was more pronounced for membranes exposed to UV radiation for a longer time and confirming the higher chemical integrity of CA (despite of the mechanical degradation observed) and NYLON membranes analyzed by FTIR analyses.

The suspensions from the experiments after UV exposure for modified membranes show considerably lower particle concentration than the unmodified membranes (Table 1 and Figure 8). These results show the protection effect promoted by the TiO_2_ layer, which was observed for all membranes except for PES-T. Previous studies evaluated the damages on different membranes caused by the photocatalytic process employing nanoparticles of TiO_2_ suspended in the aqueous medium and reported that PES was one of the most affected membranes [16]. However, in contrast with that work, the coating of the membranes with TiO_2_ nanoparticles presents an opposite effect, protecting the membranes from UV radiation. Although there were no visual changes in the color of the membrane surface (Figure S3, Supplementary Data) and the aqueous media after PES-T UV radiation exposure, the spectrum obtained by UV-Vis for PES-T (Figure 2A) denotes the presence of soluble substances released from membranes, even if in lesser quantity, suggesting a polymer degradation. The FTIR spectra for PES-T before and after UV radiation corroborated this conclusion, which can explain the lowest efficiency of *p*CBA degradation observed for the PES-T membrane (Figure S2, Supplementary Data). Probably, there is a competition between the *p*CBA molecules and the membrane polymer for the free radicals generated by UV radiation involved in the photolysis and photocatalysis processes.

Additionally, and most important, the lower concentrations of suspended particles detected by the NTA for the membranes modified with SiO_2_-TiO_2_ and exposed to 6 h to UV radiation reinforces the protection argument provided by the proposed modification, despite the cracks observed in SEM analysis. The TiO_2_ absorption ability under UV, and its chemical and physical stability, works as a UV barrier protecting the polymeric membranes against degradation by radiation [53].

Optimized and mechanized coating procedures will allow the production of more homogeneous modified membranes. Future deeper studies should address the effect of prolonged exposures to UV radiation (more than 6 h) for the specific studied membranes, associated or not with filtration, to elucidate if the light and radicals produced may affect dramatically the polymeric membranes. However, recent studies denoted that modified membranes by coating with TiO_2_ presented stability until 96 h of UV exposure [53,54].

#### 3.2.6. Water Contact Angle

**Figure 9:**
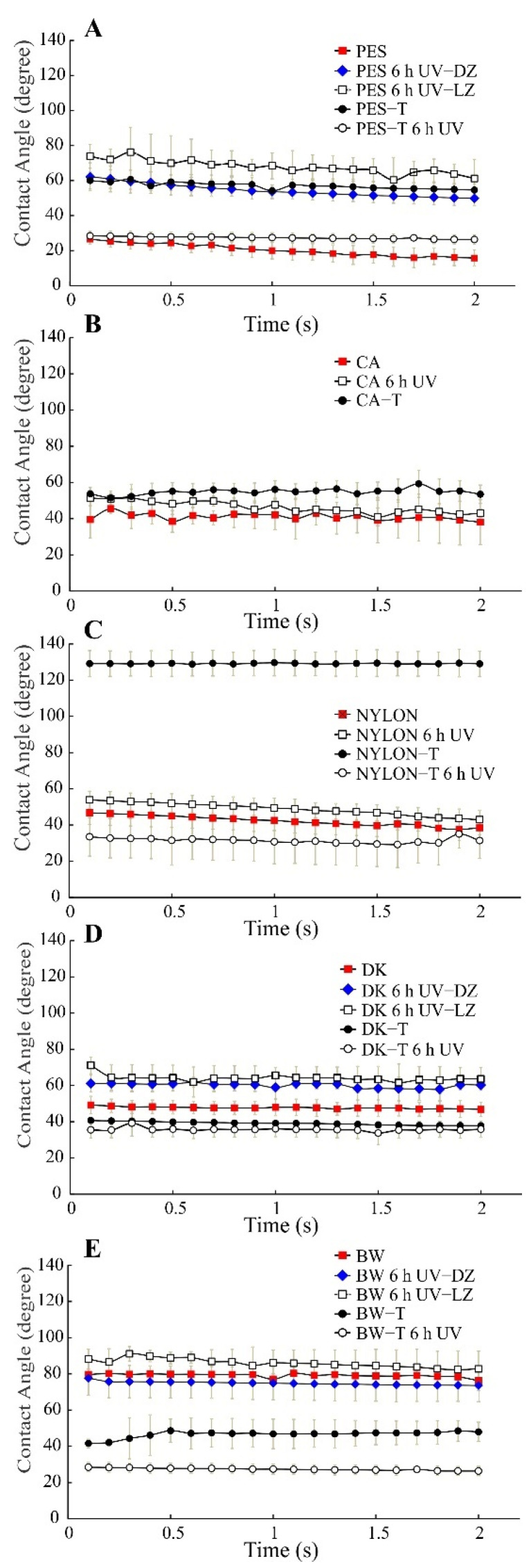
Water contact angle for unmodified and modified polymeric membranes (sol–gel modification with TiO_2_ nanoparticles) before and after UV exposure, n = 3. (**A**) PES membranes, (**B**) CA membranes, (**C**) NYLON membranes, (**D**) DK membranes and (**E**) BW membranes. Non-modified membranes: Polyethersulfone (PES, 0.2 µm), Cellulose Acetate (CA, 0.45 µm), Polyamide–Nylon (NYLON, 0.45 µm), DK and BW30; and modified membranes: PES-T, CA-T, NYLON-T, DK-T, and BW-T. DZ = dark zone, LZ = light zone. Note: For an irradiated CA-T membrane, it was impossible to measure after UV exposure due to the instantaneous water drop spreading at the membrane surface.

The contact angle is an important factor in defining the wetting ability of the membrane and is typically measured to anticipate its upcoming water permeation ability and fouling behavior [55]. Unmodified and modified membranes were tested to determine the differences of their hydrophilic character exhibited before and after 6 h of UV radiation exposure by measuring the dynamic water contact angle (Table 2 and Figure 9). For non-modified membranes, the contact angle was measured for both dark and light zones when these zones were detected.

Unmodified PES, CA, NYLON, DK, and BW membranes before UV radiation showed a dynamic contact angle between 20, 40, 40–50, 45–50, and 80 degrees. However, after sol–gel modification, the water contact angle of the modified membranes before 6 h of UV radiation exposure showed a variable behavior compared to the unmodified membranes. Thus, the water contact angle increased after TiO_2_ coating for PES-T, CA-T, and NYLON-T up to around 55–60, 53, and 130, respectively, whereas DK-T and BW-T exhibited a slight decrease (up to around 40 and 45 degrees, respectively) compared to the unmodified membranes. The water contact angle depends on the chemical composition and also on the surface morphology. Thus, differences in roughness and micro-nano structured surfaces by the deposition of TiO_2_ nanoparticles obtained after sol–gel modification evidenced in SEM images could explain the different contact angles obtained.

After 6 h UV exposure, as expected, the dynamic water contact angle of all the modified membranes decreased due to the well-known photoinduced hydrophilicity effect, being quite similar for PES-T, NYLON-T, DK-T, and BW-T, around 30 degrees (Figure 9A–D). More remarkable was the decrease in contact angle in the irradiated CA-T membrane, which is impossible to measure due to the instantaneous water drop spreading at the membrane surface. The photoinduced hydrophilicity effect was also effective in irradiated NYLON-T, DK-T, and BW-T membranes, making the drop angle values even lower than those obtained for the same unmodified polymeric membranes (Figure 9C–E). Therefore, in a filtration system coupled with UV photocatalysis, higher surface hydrophilicity is expected for the TiO_2_-modified membranes, possibly translating into a higher water permeability.

Analyzing the water contact angle for PES, DK, and BW after 6 h of exposure to UV radiation (Figure 9A,D,E), it is possible to verify changes in the hydrophilicity of the membrane surface, which can be related to chemical and morphological differences evidenced by FTIR and SEM, and the well-known effect of increasing of hydrophilicity promoted by the UV effect over TiO_2_.

### 3.3. Membrane Filtration Assays for Hormone Removal

Observing the results for removing 17α-ethinylestradiol from an aqueous medium by using the hybrid reactor without filtration, it can be concluded that there are two different processes involved in hormone removal from water for this specific set-up: (i) adsorption on the reactor surfaces and (ii) photocatalytic degradation. Considering that the rate of hormone removal by photolysis and adsorption was similar to the removal by adsorption on the surface of the hybrid reactor, it is possible to conclude that under these conditions, photolysis does not contribute significantly to reduce 17α-ethinylestradiol in the aqueous media (Figure 9A).

DK and DK-T membranes were chosen to conduct the experiments with the hybrid reactor because the molecular weight cut-off of the membrane allows the retention of 17α-ethinylestradiol. Additionally, nanofiltration membranes can be operated at lower pressure values than reverse osmosis membranes (and thus save energy), and the stability analysis showed that within the time tested, the membrane did not lead to the release of particles to the water environment (NTA results, Table 1, Section 3.2.5).

**Figure 10:**
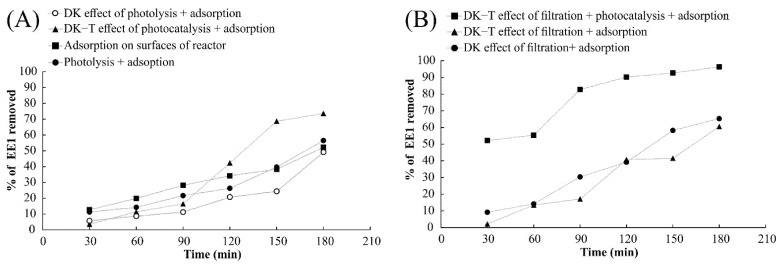
Evaluation of 17α-ethinylestradiol removal from aqueous medium by adsorption, filtration, photolysis, photocatalysis, and the combination of these processes. (**A**) Experiments without filtration and (**B**) Experiments with filtration employing DK and DK-T membranes in the hybrid reactor.

The results obtained with the DK and DK-T membranes for the filtration of 17α-ethinylestradiol resulted in very similar hormone removal, suggesting that the sol–gel coating method did not significantly affect the membrane’s filtration behavior (Figure 10B). In contrast, the combination of filtration and photocatalysis considerably increased the 17α-ethinylestradiol removal from the aqueous medium, showing that the membrane coating with TiO_2_ nanoparticles effectively reduces the hormone concentration by degrading this compound (Figure 10B). The products of degradations of 17α-ethinylestradiol by UV radiation have been proposed in the literature [56]. It is important to note that UV-Vis was also used to monitor the solution to check for any release of colored substances into the solution, which did not occur, suggesting that the coating applied provides both photocatalytic activity and membrane protection from UV exposure during the experimental time tested.

The action of the three experimental processes (adsorption, filtration, and photocatalysis) using the DK-T membrane promoted a gain of 30% in 17α-ethinylestradiol removal from the aqueous medium, leading to hormone elimination higher than 90% in 180 min of treatment. It was observed that the removal efficiency results employing photocatalysis with the DK-T membrane led to a 50% increase in 17α-ethinylestradiol removal compared to those obtained with DK and DK-T by combining adsorption in the reactor and filtration components in half the time (90 min) of the experiment, achieving removals greater than 80%. These results confirm that the TiO_2_ coating by the sol–gel method employed provides the membrane an effective photocatalytic property capable of eliminating the organic molecules present in the solution.

## 4. Conclusions

The polymeric membranes studied in this work were susceptible to degradation by exposure to UV radiation, presenting evident chemical changes observable by FTIR, the detachment of nanoparticles monitored by NTA, and the release of soluble substances detectable by UV-Vis. However, after coating with TiO_2_ nanoparticles employing a sol–gel procedure, the modified membranes acquired photocatalytic properties and protection from UV radiation. Thus, the modification of the polymeric membranes by the sol–gel coating makes the membranes less susceptible to degradation by UV exposure, with a relevant contribution of the TiO_2_ layer.

An improvement of the mechanical resistance and absence of visible alterations for the modified membranes was also confirmed. The modified membrane (DK-T) has photocatalytic potential to remove 17α-ethinylestradiol from the aqueous medium, which was confirmed in a hybrid reactor where filtration and photocatalysis occur in the same compartment, allowing to remove more than 90% of the hormone from the aqueous medium without damaging the membrane.

Future work should be conducted to improve the coating procedure in order to obtain a more homogenous TiO_2_ layer and to test the stability of the modified materials after long exposure periods to UV light.

## Figures and Tables

**Table 1:** Concentration of nanoparticles present in aqueous media for polymeric membranes not exposed and exposed to UV radiation (3 or 6 h) before and after sol–gel modification with TiO_2_. Non-modified membranes: Polyethersulfone (PES, 0.2 µm), Cellulose Acetate (CA, 0.45 µm), Polyamide–Nylon (NYLON, 0.45 µm), DK and BW; and modified membranes: PES-T, CA-T, NYLON-T, DK-T, and BW-T.

	3 h UV *	6 h UV	6 h (Dark Controls)
Membrane	Particles (10^8^)/mL		
PES	0.75	2.40	0.20
PES-T	not analyzed	0.09	0.20
DK	1.50	2.00	0.16
DK-T	not analyzed	0.03	0.04
BW	0.23	2.20	0.39
BW-T	not analyzed	0.33	0.13
CA	0.16	0.12	0.33
CA-T	not analyzed	0.01	0.02
NYLON	0.27	0.53	0.13
NYLON-T	not analyzed	0.01	0.04

* In the assays conducted with the modified membranes, samples were not collected after 3 h of UV exposure.

**Table 2:** Dynamic contact angles for unmodified and modified polymeric membranes (sol–gel modification with TiO_2_ nanoparticles) before and after UV exposure, n = 3.

	NYLON	CA	PES	DK	BW
Before UV	40–50	40	20	45–50	80
After 6 h UV	48–50	51	50–62 (DZ)	60 (DZ)	73–77 (DZ)
			61–73 (LZ)	62–70 (LZ)	82–88 (LZ)
	NYLON-T	CA-T	PES-T	DK-T	BW-T
Before UV	130	53	55–60	40	45
After 6 h UV	33	*	30	34	28

Non-modified membranes: Polyamide–Nylon (NYLON, 0.45 µm), Cellulose Acetate (CA, 0.45 µm), Polyethersulfone (PES, 0.2 µm), DK and BW30; and modified membranes: NYLON-T, CA-T, PES-T, DK-T and BW-T. DZ = dark zone, LZ = light zone. * It was impossible to measure after UV exposure due to the instantaneous water drop spreading at the membrane surface.

## Data Availability

The data presented in this study are available on request from the corresponding author.

## Supplementary Materials

The following are available online at https://www.mdpi.com/article/10.3390/polym14010124/s1, Figure S1: Hybrid reactor used in the experiments of removal of 17-α-ethinylestradiol from water employing modified membrane (DK-T), Figure S2: Efficiency of *p*CBA photocatalytic degradation employing the membranes modified with TiO_2_ (membrane*-T), Figure S3: Overview of polymeric membranes not-exposed and exposed to UV irradiation before and after sol-gel modification with TiO_2_. Non-modified membranes: Polyethersulfone (PES, 0.2 µm), Cellulose Acetate (CA, 0.45 µm), Polyamide–Nylon (NYLON, 0.45 µm), DK and BW30; and modified membranes: PES-T, CA-T, NYLON-T, DK-T, and BW-T. Membrane* identification: PES (0.2 µm Polyethersulfone), CA (0.45 µm Cellulose Acetate), NYLON (0.45 µm Polyamide-Nylon), DK and BW (BW30), Figure S4: View of the shutter of the reactor used in all experiments of membranes exposure to UV irradiation and *p*CBA degradation: A) closed; and B) opened showing the reflection of the radiation emitted by the UV-lamp, Figure S5: Overview of PES (Polyethersulfone, 0.2 µm) membranes not-exposed and exposed to UV irradiation before and after each coating layer of sol-gel modification with TiO_2_ nanoparticles, Figure S6: Aqueous media from PES membrane UV exposure denoting a release of soluble substances that turned the solution yellowish and that could be monitored by UV-Vis spectroscopy, giving a band at around 290 nm, Figure S7: FTIR of PES membrane before and after each coating layer of the procedure employed to membranes modifying with TiO_2_. The spectra are offset on the y axis to favor visualization. PES-G: 2 layers of GLYMO, PES-TEOS: PES-GLYMO + 1 layer of TEOS, and PES-T: PES-TEOS + 1 layer of TiO_2_, Table S1. Structural properties and labels of commercial membranes provided by the manufacturers, Table S2: Membranes thickness measured using a MDC-25SX Digimatic Micrometer (Mitutoyo, Japan), in at least three different random places. *t*-Test showed that the thickness of originals and modified membranes are not statistically different, Video S1: NTA for particles detached from DK, DK–T membranes after 3 and 6 h of UV exposure.
