# Supplementary material for: Stability of Polymeric Membranes to UV Exposure before and after Coating with TiO2 Nanoparticles
Source: Polymers (Basel). 2021 Dec 30;14(1):124. doi: 10.3390/polym14010124 (PMC8747730; doi:10.3390/polym14010124)
Supplement: Supplementary file 1 [file polymers-14-00124-s001.zip › polymers-1342351-supplementary.pdf]

Stability of polymeric membranes to UV exposure before and after coating with TiO_2 nanoparticles

Geórgia Labuto[a,b*], Sandra Sanches[c], João G. Crespo[d], Vanessa J. Pereira[c,e], Rosa M. Huertas[c,d]

[a]Department of Chemistry, Universidade Federal de São Paulo, Diadema - SP, 09913-030, Brazil

[b]Laboratory of Integrated Sciences – LabInSciences, Universidade Federal de São Paulo, Diadema - SP, 09913-030, Brazil

[c]iBET, Instituto de Biologia Experimental e Tecnológica, Apartado 12, 2781-901 Oeiras, Portugal

[d] LAQV-REQUIMTE, Department of Chemistry, NOVA School of Science and Technology, FCT NOVA, Universidade NOVA de Lisboa, 2829-516 Caparica, Portugal

[e]Instituto de Tecnologia Química e Biológica António Xavier, Universidade Nova de Lisboa, Av. da República, 2780-157 Oeiras, Portugal

* *Correspondent author*: geolabuto@gmail.com

(geolabuto@gmail.com) Geórgia Labuto, (rosa.huertas@ibet.pt) Rosa M. Huertas, (sandras@ibet.pt) Sandra Sanches, (vanessap@ibet.pt) Vanessa J. Pereira, (jgc@fct.unl.pt) João G. Crespo

1. Development of photocatalytic membranes

Two layers of 3-(Glycidyloxypropyl)trimethoxysilane (GLYMO) were firstly deposited in the first step (S1) to promote a compatible surface between the polymeric structure and the subsequent inorganic layers. The GLYMO is well-known to be a good epoxy-functionalized organic-inorganic SiO_2 precursor and acts as a compatible interface layer between the polymer and hydroxyl groups present in the subsequent layers to be deposited [A,B]. To prepare the solution used in S1, 2 mL of GLYMO (Aldrich, Germany) and 2 mL of acetic acid glacial (Sigma Aldrich, Germany) were diluted in 500 mL of water and stirred for 2 hours for hydrolysis (Figure 1).

A SiO_2 precursor solution of tetraethyl orthosilicate (TEOS-1) was prepared to obtain the solutions employed in steps S2 and S3. The solution TEOS-1 was prepared by dissolving 29.2 mL of TEOS (98%, Sigma Aldrich, Germany) in 5.8 mL ethanol (EtOH, Merck, Germany) and 7.2 mL of distilled water and stirred for 30 min (250 rpm). Subsequently, 0.03 mL concentrated HCl (37% Merck, Germany) was added to catalyze the hydrolysis (60 min, 250 rpm). After that, an aliquot of 10 mL of TEOS-1 was diluted

in absolute EtOH yielding a total volume of 200 mL which was stirred at ambient temperature (24 h) and used to perform the TEOS coating in step 2 (S2).

A TEOS-2 solution was obtained by mixing 2.22 mL of TEOS-1 + 2.92 mL of EtOH + 0.040 mL of HCl, and 0.72 mL of distilled water (molar ratio 1:5:0.05:4, respectively) [A,C]. The TEOS-2 solution was stirred (1 h, 250 rpm) to ensure complete hydrolysis. This solution was used to prepare the SiO_2-TiO_2 solution used in step 3 (S3) by mixing 2 g of the commercially available Degussa P25 titanium dioxide nanoparticles (30–90 nm of nominal diameter; Nanoshell, England; Transmission electronic microscopy images and particle size distribution available in Table S1 of the Supplementary Data section) in 13.4 mL TEOS-2 and diluting to 500 mL with EtOH (Merck, Germany). The final SiO_2:TiO_2 molar ratio composition was 0.9:1, being the concentration 0.045 mol/L of SiO_2 and 0.050 mol/L of TiO_2. The coverage and drying of the membranes were performed using a filtration system (Nalgene PS 250 mL, 45 mm diameter) as a support to keep the membranes flat during the process.

References:

A. *Fateh, R.; Dillert, R.; Bahnemann, D. Preparation and Characterization of Transparent Hydrophilic Photocatalytic TiO2/SiO2 Thin Films on Polycarbonate, Langmuir 2013, 29, 3730-3739. https://doi.org/10.1021/la400191x.*

B. *Adachi, T.; Latthe, S.S.; Gosavi, S.W.; Roy, N.; Suzuki, N.; Ikari, H.; Kato, K.; Katsumata, K.; Nakata, K.; Furudate, M.; Inoue, T.; Kondo, T.; Yuasa, M.; Fujishima, A.; Terashima, C. Photocatalytic, superhydrophilic, self-cleaning TiO2 coating on cheap, light-weight, flexible polycarbonate substrates, Appl. Surf. Sci. 2018, 458, 917-923. https://doi.org/10.1016/j.apsusc.2018.07.172*

C. *Huertas, R.M.; Fraga, M.C.; Crespo, J.G.; Pereira, V.J. Sol-gel membrane modification for enhanced photocatalytic activity. Sep. Pur. Technol. 2017, 180, 69-81. http://dx.doi.org/10.1016/j.seppur.2017.02.047.*

2. Description of instruments and conditions employed for characterization of membranes before and after UV radiation exposure

2.1. Fourier transform infrared (FTIR) spectroscopy analysis

The chemical structure of the unmodified and modified membranes before and after UV radiation exposure was analyzed by Fourier transform infrared spectroscopy (FTIR) with attenuated total reflectance (ATR). A Bruker Spectrometer IFS 66/S

instrument (USA) equipped with an H-ATR and a ZnSe crystal was used to evaluate the chemical stability of the membranes by analyzing the presence of the main functional groups. The membrane samples were dried overnight in a desiccator at room temperature and analyzed in different random positions to check the homogeneity of their chemical composition. The normalized spectra were recorded in the range of wavenumbers from 4000 to 550 cm^{-1} during 20 scans with a resolution of 4 cm^{-1}.

2.2. Scanning electron microscopy (SEM)

The morphology (top and cross-section) of the unmodified and modified membranes were analyzed by scanning electron microscopy (SEM) and energy dispersive X-ray spectrometry (EDS) mapping analysis. Morphology (top and cross-section) of the unmodified and modified membranes were analyzed by scanning electron microscopy (SEM) and energy dispersive X-ray spectrometry (EDS) mapping analysis. The membranes were cut carefully under liquid nitrogen, and the membrane surface was sputter-coated with an Au/Pd thin film using a South Bay E5100 apparatus. They were then analyzed by scanning electron microscopy in a JEOL FEG-SEM model JSM-7001 microscope.

2.3. Water contact angle of membranes before and after UV radiation exposure

The hydrophilicity of the unmodified and modified membranes before and after UV exposure was evaluated by measuring the dynamic water contact angle [**Error! Reference source not found.**] by the sessile drop method using A KSV CAM2008 equipment. A distilled water drop (10-12 μL) was used to obtain the contact angle on at least three different places of membranes randomly chosen. For membranes that presented changes in color after UV exposure, the contact angle was measured in the light zone (LZ) and dark zone (DZ). The contact angles were measured using automatic image analysis. Twenty frames were attained for each measurement with a frame interval of 100 ms.

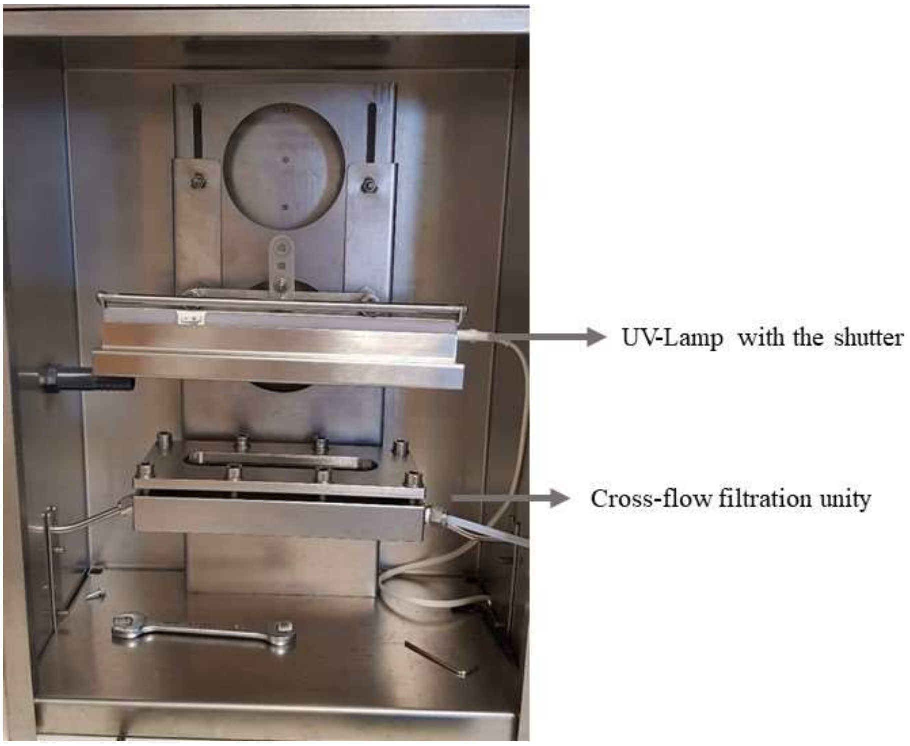

Figure S1. Hybrid reactor used in the experiments of removal of 17-α-ethinylestradiol from water employing modified membrane (DK-T).

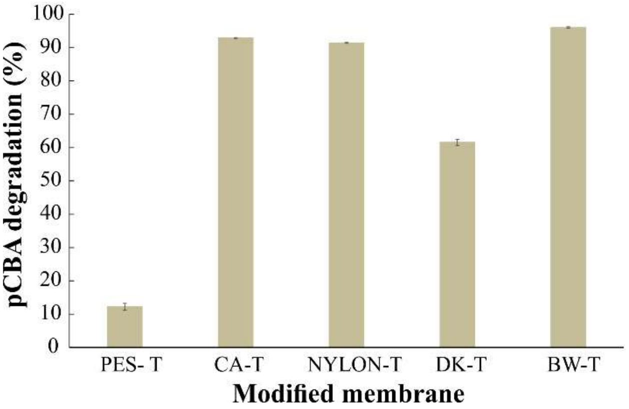

Figure S2. Efficiency of *p*CBA photocatalytic degradation employing the membranes modified with TiO_2 (membrane*-T). Membrane* identification: PES (0.2 µm Polyethersulfone), CA (0.45 µm Cellulose Acetate), NYLON (0.45 µm Polyamide-Nylon), DK and BW (BW30).

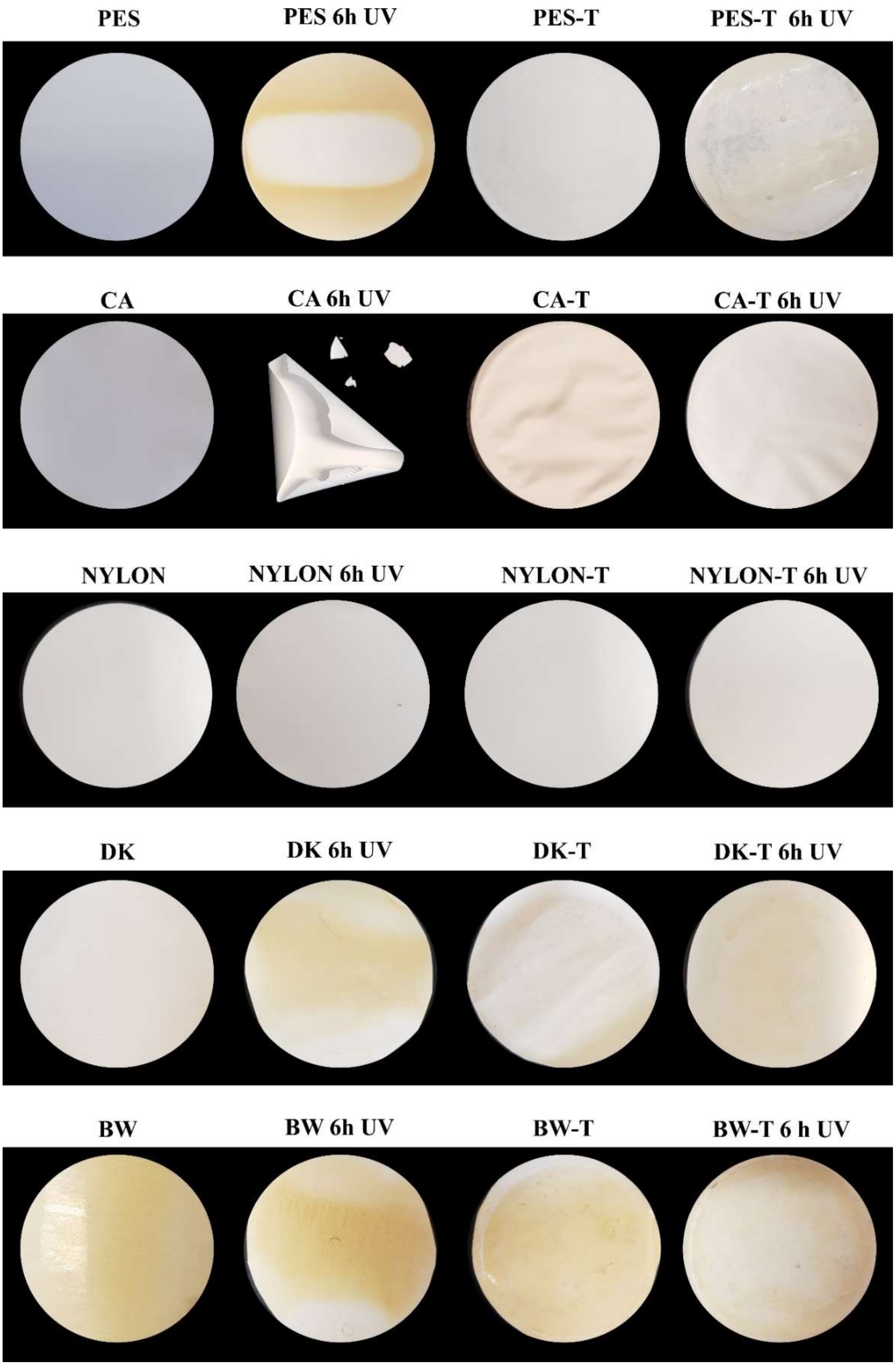

Figure S3. Overview of polymeric membranes not-exposed and exposed to UV irradiation before and after sol-gel modification with TiO_2. Non-modified membranes: Polyethersulfone (PES, 0.2 μm), Cellulose Acetate (CA, 0.45μm), Polyamide – Nylon (NYLON, 0.45μm), DK and BW30; and modified membranes: PES-T, CA-T, NYLON-T, DK-T, and BW-T.

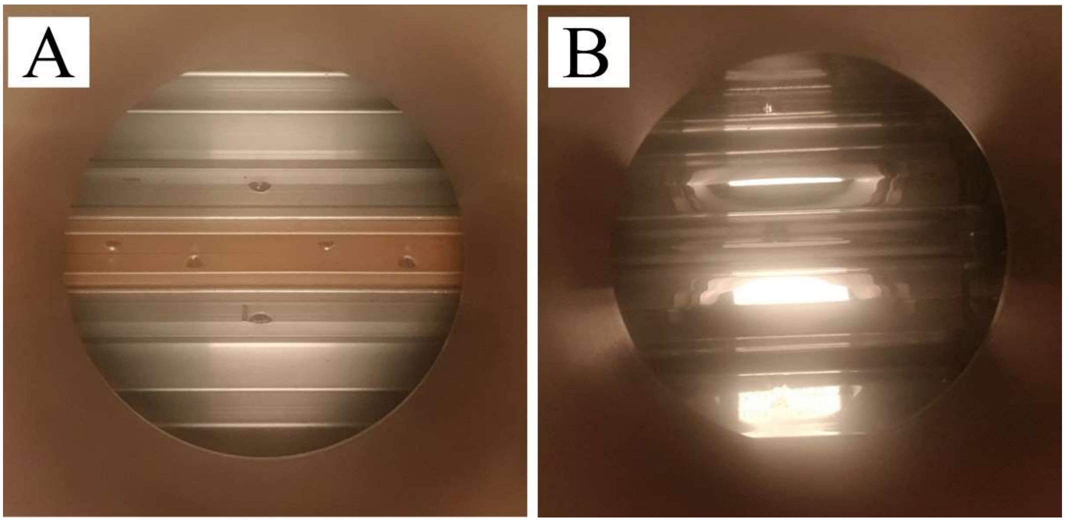

Figure S4. View of the shutter of the reactor used in all experiments of membranes exposure to UV irradiation and pCBA degradation: A) closed; and B) opened showing the reflection of the radiation emitted by the UV-lamp.

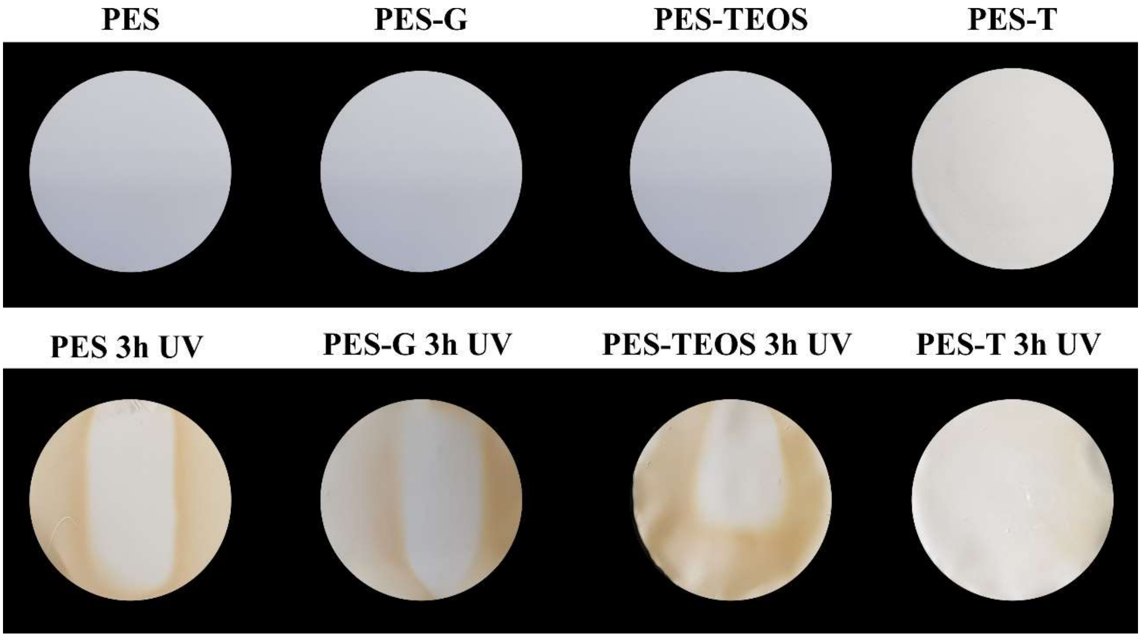

Figure S5. Overview of PES (Polyethersulfone, 0.2 μm) membranes not-exposed and exposed to UV irradiation before and after each coating layer of sol-gel modification with TiO_2 nanoparticles.

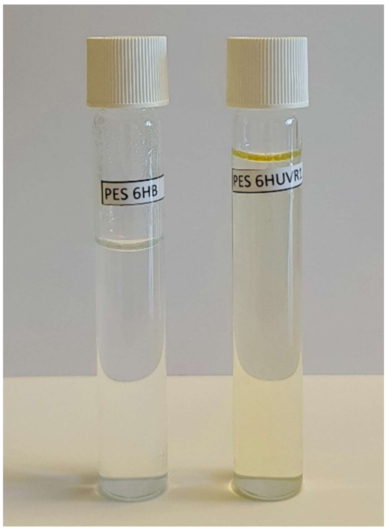

Figure S6. Aqueous media from PES membrane UV exposure denoting a release of soluble substances that turned the solution yellowish and that could be monitored by UV-Vis spectroscopy, giving a band at around 290 nm.

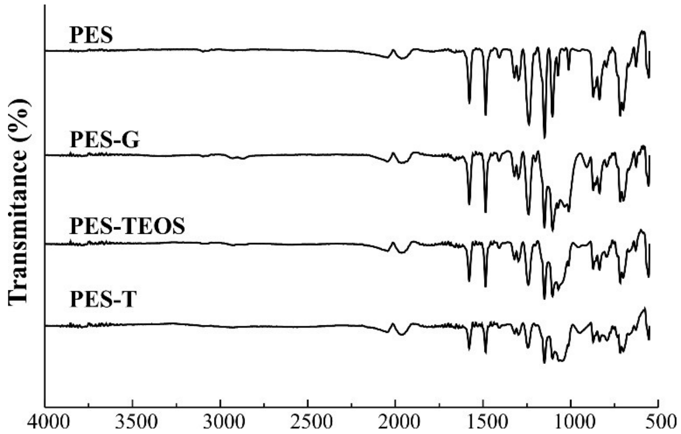

Figure S7. FTIR of PES membrane before and after each coating layer of the procedure employed to membranes modifying with TiO_2. The spectra are offset on the y axis to favor visualization. PES- G: 2 layers of GLYMO, PES-TEOS: PES-GLYMO + 1 layer of TEOS, and PES-T: PES-TEOS + 1 layer of TiO_2.

Table S1. Structural properties and labels of commercial membranes provided by the manufacturers.

Sample	Water Flow rate and/or Permeability	molecular weight cut-off	Thickness (μm)
Polyethersulfone 0.2 μm (PES) (Gelman Sciences, USA) *	26mL/min/cm2 at 0.7 bar (70 kPa, 10 psi)		145
Polyamide-Nylon 0.2 μm (NYLON) (Whatman GE, USA)**	> 50 mL/min (5 psi)		150-187
Cellulose Acetate 0.45 μm (CA) (Whatman, Japan) ***	12 at $\Delta p = 0.9$ bar (s/100 ml/12.5 cm^2)		115
Polyamide Thin-Film Composite membranes DK (GE, USA) ****	20 (L/h*m^2) at 20 °C and 3×10^5 Pa	150-300 Da	
BW30-400 (BW) (Dupont, FilmtecTM Membranes, USA)	24.2 L/m^2-hr, at 15.4 bar 40 (m^3/d); Permeate flow and salt (NaCl) rejection based on the following standard test conditions: 2,000 ppm NaCl, (15.5 bar), (25°C), pH 8, 15% recovery. For pure water: 2.3 ± 0.7 (L.h-1.m^{-2}.bar^{-1})	90 Da	

Website references:

* https://www.pall.com/content/dam/pall/laboratory/literature-library/non-gated/microporous-membranesanddiscfiltersforenvironmentalwaterqc/Supor%C2%AE%20PES%20Membrane%20Disc%20Filters%20.pdf

**https://beta-static.fishersci.com/content/dam/fishersci/en_EU/suppliers/Whatman/Whatman_Lab_Filtration_Product_Guide2.pdf

*** http://www.mar-con.cz/cenik/Whatman_Catalog.pdf

**** The use of nanofiltration membranes for the fractionation of polyphenols from grape pomace extracts; DOI : 10.20870/oeno-one.2018.52.4.1580

***** https://pureaqua.com/content/pdf/dow-filmtec-bw30-400-membrane.pdf; https://tel.archives-ouvertes.fr/tel-00433513/document

Table S2. Membranes thickness measured using a MDC-25SX Digimatic Micrometer (Mitutoyo, Japan), in at least three different random places. t-Test showed that the thickness of originals and modified membranes are not statistically different.

Sample	Average Thickness (mm)
PES	0.146±0.001
PES-T	0.147±0.002
DK	0.137±0.002
DK-T	0.136±0.006
BW	0.141±0.005
BW-TiO_2	0.145±0.007
NYLON	0.176±0.001
NYLON-T	0.181±0.001
CA	0.127±0.004
CA-T	0.141±0.012

2.4. Information about Degussa P25 titanium dioxide nanoparticles

Source: https://www.nanoshel.com/product/degussa-p25-titanium-dioxide/

Degussa p25 Titanium Dioxide Nanopowder

Daiki-200®

(TiO_2, Degussa, Rutile:Anatase/ 85:15, 99.9%, 20nm)

Available Pack Size: 10Gms, 25Gms, 50Gms, 100Gms, 250Gms, 500Gms, 1Kg & Bulk orders

Like

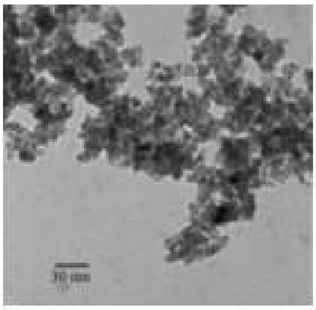

TEM - TiO2 Nanopowder

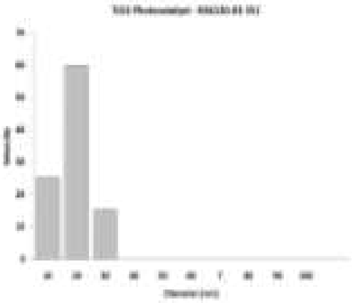

Particles Size Analysis

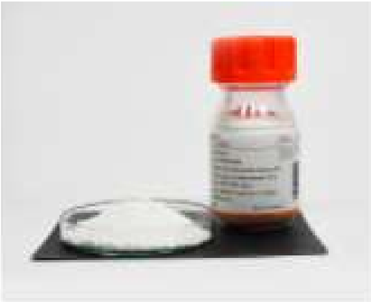

Titanium Dioxide

**Product**	**Degussa p25 Titanium Dioxide Nanopowder**	
Stock No	NS6130-03-351	
CAS	13463-67-7	Confirm
HS Code	32061110	Confirm
Purity	99.9 %	Confirm
APS	20nm	Confirm
Rutile	85%	Confirm
Anatase	15%	Confirm
Molecular Formula	TiO2	Confirm
Molecular Weight	79.87 g/mol	Confirm
Form	Powder	Confirm
Color	White	Confirm
Density	4.26 g/cm^3	Confirm
Solubility	Insoluble in water, dilute acids, organic acids.	
**Quality Control**	Each lot of **Degussa p25 Titanium Dioxide Nanopowder** was tested successfully.	
**Main Inspect Verifier**	**Manager QC**	

Typical Chemical Analysis

Assay	99.9 %
Other Metal	<1000ppm
